# Duodenal Hemorrhage Due to an Invasive Hepatocellular Carcinoma Controlled by Transarterial Embolization

**DOI:** 10.7759/cureus.32046

**Published:** 2022-11-30

**Authors:** Maxime Deforche, Ana-Maria Bucalau, Illario Tancredi, Fadi Tannouri, Gontran Verset

**Affiliations:** 1 Department of Radiology/Interventional Radiology, Hôpital Erasme/Université Libre de Bruxelles, Brussels, BEL; 2 Department of Gastroenterology, Hepatopancreatology and Digestive Oncology, Hôpital Erasme/Université Libre de Bruxelles, Brussels, BEL

**Keywords:** hepatoduodenal fistula, immunotherapy, chemotherapy, transarterial embolization, gastro-intestinal bleeding, hepatocellular carcinoma

## Abstract

Upper gastrointestinal (GI) bleeding due to duodenal invasion is a very unusual presentation revealing the initial diagnosis of hepatocellular carcinoma (HCC), especially in patients without cirrhosis. No clear recommendations are available in this setting. A 68-year-old man was admitted to the emergency department with melena. The esophagogastroduodenoscopy (EGD) revealed an oozing hemorrhagic ulcer of the duodenal bulb (Forrest I b) secondary to an invasive, undetermined bulky liver mass that was biopsied. The histopathological examination confirmed an HCC. The patient was started on chemotherapy (Gemcitabine and Oxaliplatin) with good initial response. Nevertheless, after eight months of treatment, there was a recurrence of the ulcer bleeding and a disease progression was identified. Selective transarterial embolization (TAE) was used to control the duodenal bleeding, permitting the patient to receive immunotherapy with a long-lasting control of the disease. Our case report suggests that selective TAE is a therapeutic option that can be used to stop GI bleeding due to invasive HCC in order to allow oncological treatment.

## Introduction

Hepatocellular carcinoma (HCC) is the main type of primary liver cancer and arises typically in patients with liver cirrhosis. HCC is the third leading cause of cancer-related deaths worldwide and the sixth most common cancer [1]. Incidence is still increasing, with men being more affected than women, it is highly prevalent in Asia and the incriminated factors are identical to those of cirrhosis (chronic hepatitis B or C, excessive alcohol use and nonalcoholic fatty liver disease [NAFLD]) [1,2]. When occurring in the context of cirrhosis, diagnosis could be made by noninvasive criteria, using contrast-enhanced MRI (CE-MRI) or contrast-enhanced computed tomography (CECT), according to the European Association for the Study of the Liver/American Association for the Study of Liver Diseases guidelines [1,2]. For patients without cirrhosis, the diagnosis of HCC is often made in a more advanced stage, when patients develop symptoms, and histopathological confirmation is required. The most common symptoms described in patients with HCC are lack of appetite, weight loss, and abdominal pain, associated with clinical signs of liver decompensation (jaundice, ascites, and encephalopathy). Gastrointestinal bleeding is usually secondary to portal hypertension associated with cirrhosis or tumor invasion of the portal vein, but is very rarely due to direct gastrointestinal tumor invasion. Based on the Barcelona Clinic Liver Cancer (BCLC) classification, the standard of care is transarterial chemoembolization (TACE) for intermediate-stage and systemic therapy for advanced-stage HCC [1,2]. Transarterial embolization (TAE) is achieved by injecting small particles, with (TACE) or without (TAE) chemotherapy, to occlude hepatic arterial supply of the tumor resulting in ischemia and necrosis. Here we report the case of a patient presenting multifocal HCC diagnosed after the occurrence of a gastrointestinal hemorrhage due to a duodenal invasion by one of the HCC nodules. As there are no specific guidelines in the literature for the management of this type of bleeding, selective TAE was used to control the duodenal bleeding, permitting the patient to receive sequential treatment by immunotherapy with a long-lasting control of the disease.

## Case presentation

**Figure 1:**
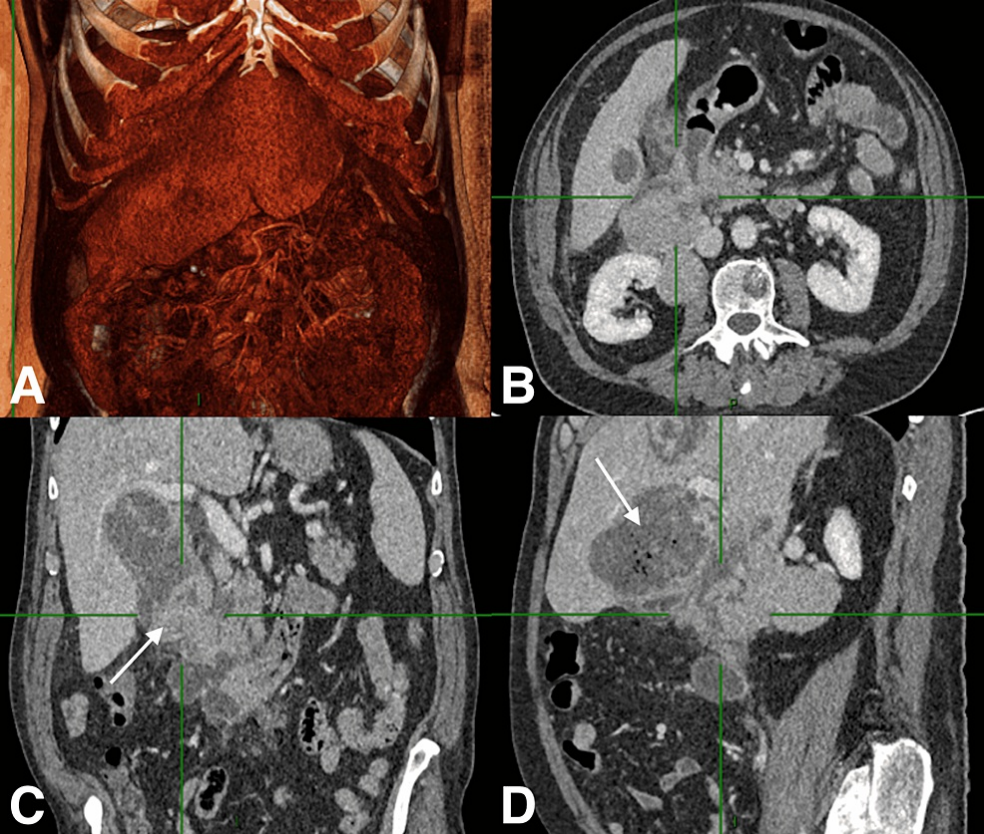
CECT with an invasive duodenal HCC nodule CECT, one week after the first TAE session, showed an invasive duodenal HCC nodule with persistent hypervascularization (white arrow in C) and tumor necrosis (white arrow in D) displayed with (A) VRT, (B) axial, (C) coronal and (D) sagittal planes. CECT: Contrast-enhanced CT; TAE: Transarterial embolization; HCC: Hepatocellular carcinoma; VRT: Volume rendering technique.

A 68-year-old man was admitted to the emergency department with melena and the diagnosis of an upper gastrointestinal bleeding was suspected. Within 24 hours the patient underwent an esophagogastroduodenoscopy (EGD) which revealed an oozing hemorrhagic ulcer at the duodenal bulb (Forrest I b) secondary to an invasive undetermined bulky tumor mass which was biopsied. The bleeding was treated with intra-tumoral adrenalin injection, argon plasma coagulation and endoscopic hemostatic clips. A complementary CECT and histopathological examination confirmed multinodular HCC with duodenal invasion developed due to an underlying non-alcoholic fatty liver disease (NAFLD) without cirrhosis. The patient was not eligible for curative treatment such as surgical resection or ablation, and a systemic chemotherapy by the association of Gemcitabine and Oxaliplatin (GEMOX) was decided during the multidisciplinary team (MDT) in order to avoid duodenal necrosis or bleeding which could be induced by TACE or tyrosine kinase inhibitors. A stable disease was obtained with GEMOX during eight months until the patient presented a recurrence of upper gastrointestinal (GI) bleeding and control EGD identified local disease progression. He was referred to our tertiary center where we decided to treat the nodule responsible for the duodenal hemorrhage by selective trans-arterial embolization without chemotherapy (TAE). For the first TAE session, we injected cautiously and very selectively bland particles of 200 μm (4 ml) in the feeding arteries of the invading HCC nodule. The portion of the tumor invading the duodenal wall was vascularized by both hepatic arteries and duodenal branches. In order to avoid mucosal necrosis, these duodenal branch arteries were occluded by micro-coils. After the first TAE, the EGD showed shrinkage of the nodule in the duodenal lumen with persistent slight bleeding and CECT confirmed the persistence of nodule hypervascularization with partial tumor necrosis (Figure 1B-1D).

**Figure 2:**
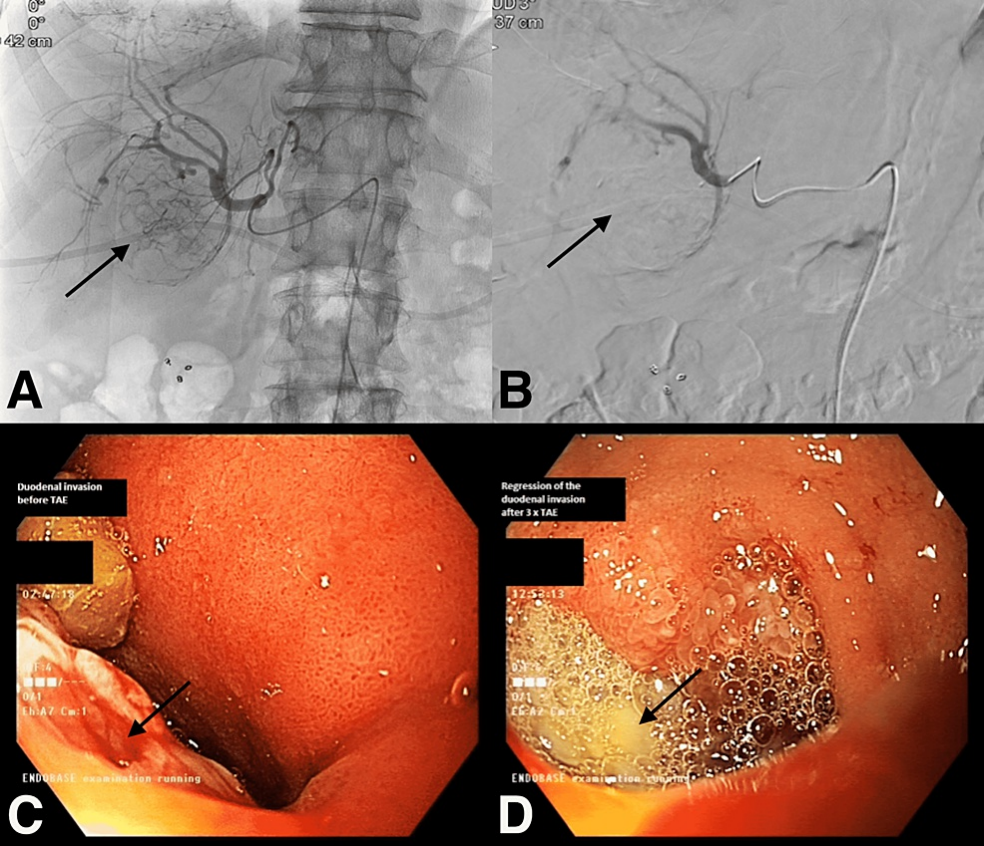
Angiographic and endoscopic imaging features of the HCC tumor (A) Fluoroscopy-guided angiography image before embolization with a delineated hyper-vascular HCC tumor with numerous feeding arteries (black arrow). (B) Digital subtraction angiography (DSA) image after embolization showing marked devascularization of the target tumor and a reduced number of feeding arteries (black arrow). (C) Endoscopy before TAE demonstrating duodenal stenosis due to intraluminal invasion by a hyper-vascular HCC tumor with active bleeding (oozing - black arrow). (D) Endoscopy after third session of TAE showing resolution of the stenosis and bleeding but persistence of clear base ulcer (black arrow). HCC: Hepatocellular carcinoma; DSA: Digital subtraction angiography; TAE: Transarterial embolization.

These results led to a complementary second session of TAE, with similar particles. Both TAE sessions were well tolerated by the patient without side effects and control EGD demonstrated complete bleeding control. At that time, immunotherapy with Atezolizumab was started and stable disease was obtained during one year, according to RECIST 1.1 criteria. Recurrence manifested by upper GI bleeding and EGD showed active hemorrhage (oozing) and enlargement of the tumor occupying two-thirds of the duodenal circumference (Figure 2C). The other lesions were stable according to CECT. This led us to carry out a third TAE session which was more aggressive using small and large size particles (from 200 - 500 μm) until contrast stasis in the feeding arteries of the tumor (Figure 2A-2B).

This session intended both hemostatic treatment and sharply decreasing tumor size. Control EGD performed after one week showed a significant reduction in the size of the sub-occlusive tumor. Moreover, there was no more bleeding (Figure 2D). A two weeks’ control CECT scan disclosed a fluid collection in contiguity with the bulky duodenal tumor mass probably due to necrosis and noted as a mild adverse event (grade 1) according to the CTCAE (common terminology criteria for adverse events) accepted by CIRSE (cardiovascular and interventional radiological society of Europe). He had no re-bleeding ever since and is still under treatment by immunotherapy with an excellent quality of life.

## Discussion

Gastrointestinal bleeding due to duodenal invasion is a very unusual presentation revealing the initial diagnosis of HCC, especially in patients without cirrhosis. Frequently, bleeding is due to variceal rupture caused by portal hypertension. Upper gastrointestinal HCC invasions are presented in the form of melena, anemia, hematemesis, upper GI obstruction and abdominal pain [3]. Gastrointestinal involvement by metastatic or continuous malignancy is a rare condition and accounts for only 0.5-2% of the HCC cases [4,5]. A prospective study in an Asian population with known HCC and either hematemesis or melena, showed an incidence of only 5% of hemorrhage due to direct tumor invasion [6].

Since the first case report in 1987 by Humbert et al. [7], only a few cases of bleeding due to invasive duodenal HCC were described in the literature. To our best knowledge, the largest series reported 21 cases of duodenal involvement by metastatic or continuous HCC with GI bleeding in 18 patients with a mean survival of 10.5 months [8]. Chen et al. suggested that GI invasion was mostly seen in patients after TAE or TACE due to a reactive inflammatory response in the nearby intestinal wall causing adherence to the tumor capsule [5,9,10]. However, this notion was contested by Park et al. arguing other etiologies like growth pattern, size and tumor location [3,11].

In our patient, all TAE sessions were performed after the diagnosis of GI invasion and helped to reduce and, in the end, stop the bleeding. TACE, the standard treatment for intermediate-stage HCC (BCLC-B), is performed for the treatment of large unresectable HCC noneligible for other treatments or prior to liver transplantation. The median survival with TACE is 26 months, and beyond 40 months in selected BCLC-B patients [1]. The management of variceal bleeding is well defined and frequently updated during the Baveno consensus workshop [12], whereas there are no established recommendations for gastrointestinal bleeding due to direct HCC tumor invasion. Different hemostatic treatments for this type of bleeding have been reported in the literature, like endoscopic hemostasis, TAE, surgery or radiotherapy [9,13-18]. In addition, De Somer et al. reported the first case of a hepatoduodenal fistula due to a multifocal HCC successfully treated by immunotherapy (nivolumab) [19]. Even if some authors consider surgery as the best option to stop GI bleedings in patients with HCC invading the duodenum, only a few cases of resection are reported in the literature. A review by Kato et al. presented four surgical cases in which only two patients were eligible for major surgery like hepatectomy with pancreaticoduodenectomy (HPD) whereas Ito et al. proposed a successful less invasive procedure, more precisely hepatectomy with pancreas-preserving partial duodenectomy (HPPD), in vulnerable patients [13,14]. However, these highly invasive procedures need to be carefully evaluated and rarely recommended in HCC patients that already present an underlying liver disease and are usually in a poor general condition. In our patient, TAE was demonstrated to be very useful in controlling active bleeding and reducing the mass effect due to tumor invasion. Another beneficial argument, not to be underestimated, is the preservation of the patient’s quality of life due to a mini-invasive procedure. Moreover, the patient was able to continue systemic treatment thanks to successful management of the GI bleeding by TAE. He is currently at more than 22 months from initial diagnosis. A close follow-up, with CECT and EGD, is important in order to avoid duodenal obstruction.

## Conclusions

Duodenal invasion with upper GI bleeding is an unusual presentation for HCC. Our case report suggests that selective TAE is a valuable therapeutic option that can be used to control GI hemorrhage in order to allow oncological treatment.
